# Infective endocarditis and diabetes mellitus: Results from a single-center study from 1994 to 2017

**DOI:** 10.1371/journal.pone.0223710

**Published:** 2019-11-18

**Authors:** Rossella M. Benvenga, Roberta De Rosa, Angelo Silverio, Rosanna Matturro, Cristina Zambrano, Alfonso Masullo, Generoso Mastrogiovanni, Lucia Soriente, Roberto Ascoli, Rodolfo Citro, Federico Piscione, Gennaro Galasso

**Affiliations:** 1 Department of Medicine, Surgery and Dentistry, University of Salerno, Salerno, Italy; 2 Heart Department, University Hospital “San Giovanni di Dio e Ruggi d’Aragona”, Salerno, Italy; Ziekenhuisgroep Twente, NETHERLANDS

## Abstract

**Background:**

To evaluate the prognostic impact of diabetes mellitus (DM) in patients with Infective Endocarditis (IE).

**Methods and results:**

375 patients with diagnosis of IE referred to our Hospital between 1994–2017 were retrospectively included; diabetes was reported in 129 (34.4%). Diabetic patients were older than non-diabetic (66±1 vs. 57±2 years, p<0.001) and showed a higher prevalence of comorbidities such as hypertension (75 vs. 54%, p<0.001), coronary artery disease (30 vs. 12%, p<0.001) and history of heart failure (HF; 24 vs. 13%, p = 0.021). Echocardiography showed a higher incidence of paravalvular complications (82 vs. 64%, p<0.001) and a lower left ventricular ejection fraction (LVEF; 52±11 vs. 55±10%, p = 0.001) in diabetic than in non-diabetic patients. In-hospital mortality was higher in diabetic patients (83 vs. 74%; p = 0.030). At logistic regression, history of HF (OR = 3.1, 95%CI: 1.87–5.29, p<0.001) resulted an independent predictor of in-hospital death.

At long-term follow-up [median 24(7–84) months], the Kaplan-Meier analysis showed a significantly lower survival free from all-cause death in the group with diabetes (Log-rank<0.001). At the propensity score adjusted Cox multivariable analysis, DM (HR = 1.76, 95%CI: 1.18–2.6, p = 0.005), age (HR = 1.03, 95%CI: 1.02–1.05, p<0.001), intravenous drug users (HR = 5.42, 95%CI: 2.55–11.51, p<0.001) and low LVEF (HR = 0.98, 95%CI: 0.96–0.99, p = 0.013) were independently associated to a higher mortality.

**Conclusion:**

In patients with IE, DM is associated to a higher prevalence of anatomic complications and a more impaired LVEF. Diabetic patients show a significantly lower survival both in hospital and during follow-up compared to the non-diabetic ones.

## Introduction

Infective Endocarditis (IE) is a challenging clinical entity associated with a high in-hospital mortality ranging from 10 to 26%, and an estimated 5-years survival of 60–70% [1]. Despite the effort in the development of evidence-based prophylactic strategies [2], the incidence of this life-threatening disease has not been significantly reduced in the recent years [3]. Furthermore, the development of new and more sophisticated diagnostic tools for isolation of microorganism, characterization of vegetation and identification of complication, as well as the possibility of a new pharmacological or surgical antimicrobial therapy, have only slightly modified the natural history of patients suffering from IE [4–7]. Continuous microbiological and epidemiological changes can be responsible for this persistently poor prognosis. Indeed, whereas in the past IE was predominantly caused by community-acquired, Streptococci Viridans, today the most common isolated pathogenic microorganisms are Staphylococci [8]. Moreover, epidemiology of IE patients has changed over the years: while in the past patients presenting with IE were mainly young, with rheumatic or congenital valve disease, currently the majority of IE patients are elderly, frail, with several comorbidities, raising many problems in daily clinical management and influencing the outcome [9–10]. In this scenario, the prevalence of diabetes mellitus (DM) among patients with IE has markedly increased over time [11]. Indeed, patients with DM have a high risk of infections due to many acquired immunological dysfunctions (i.e. depressed leukocyte chemotaxis and adherence, reduced phagocytosis and impaired antioxidant systems) [12]. Moreover, diabetic patients with infective diseases generally show increased clinical severity and poorer outcome compared with non-diabetic patients.

To date, the prognostic impact of DM in terms of morbidity and mortality in patients with IE remains unclear. We aimed to characterize the clinical, echocardiographic and microbiological features of diabetic patients with IE and to investigate whether DM might influence the short and long-term clinical outcome of patients with IE.

## Methods

### 2.1 Study population

We retrospectively collected the medical records of all patients with definite diagnosis of IE admitted from April 1994 to April 2017 at our institution. The diagnosis of IE was based on the “von Reyn criteria” until 1994 and on the “Duke” and “modified Duke criteria” thereafter, according to the current scientific recommendation [13–15]. DM was diagnosed according to the American Diabetes Association criteria if the patient had a fasting glucose ≥126 mg/dL or a glycated hemoglobin (HbA1c) ≥6.5% if taking hypoglycemic drugs [16]. All demographic, clinical, laboratory and echocardiographic data were collected and stored in a dedicated database. The study was approved by the local ethics committee (Campania Sud), which waived the requirement for informed consent. The investigation conforms with Declaration of Helsinki principles.

### 2.2 Echocardiography

All patients underwent transthoracic echocardiography (TTE), whereas transesophageal echocardiography (TEE) was performed according to specific clinical setting [6]. All the echocardiographic exams were performed during the acute phase of IE. The following parameters were collected for all patients: left ventricular ejection fraction (LVEF), vegetation morphology, infective involvement of native/prosthetic valves or intracardiac device, valvular regurgitation and/or obstruction, morphological features (vegetation, abscess, dehiscence of prosthetic valve) and paravalvular complications of IE (fistula, pseudoaneurysm, perforation, valve aneurysm), if anything [17]. Morphological features and complications of IE were characterized by echocardiography according the current consensus. Briefly, a vegetation was identified as an oscillating or non-oscillating intracardiac mass on valve surface or other endocardial structures, or on implanted intracardiac devices; it was subsequently characterized according to size, mobility and extent as according with criteria of Sanfilippo et al [18]: to the size ≤6 mm (grade 1), 7–10 mm (grade 2), 11–15 mm (grade 3), >15 mm (grade 4); to the mobility: a fixed vegetation without no detectable independent motion (grade 1), with a fixed base but with a mobile free edge (grade 2), a pedunculated vegetation, defined as a vegetation with a greater perpendicular dimension than its parallel dimension, but that remains within the same chamber throughout the cardiac circle (grade 3), a prolapsing the coaptation plane of the leaflets during the cardiac circle (grade 4); to the extention: a single vegetation (grade 1), multiple vegetations limited to a single valve leaflet (grade 2), involvement of multiple valve leaflets (grade 3), a vegetation that extended to extra-valvular structures (grade 4).

Other anatomical and echocardiographic features were defined according the current recommendations of the European association of echocardiography [17]. An abscess was identified as a thickened, non-homogeneous paravalvular area with echo-dense or echo-lucent appearance; a pseudoaneurysm was described in case of pulsatile paravalvular echo-free space, with flow detected by color-Doppler; a perforation was identified by an interruption of endocardial tissue continuity traversed by flow at color-Doppler; a fistula was described in the presence of a flow-communication between two neighboring cavities through a perforation; a valve aneurysm and a prosthetic valve dehiscence were identified by a saccular bulging of valvular tissue and by a paravalvular regurgitation of a prosthesis, respectively.

### 2.3 Microbiology

The samplings for microbiological analysis were collected prior to initiation of antibiotic therapy. The results of blood culture were reported according to the followings categories: oral Streptococci (formerly Viridans), group D Streptococci, Coagulase-negative Staphylococci (CoNS), Staphylococcus Aureus, Candida and others.

### 2.4 Clinical outcome

The primary study outcome was the occurrence of death for any cause at the longest available follow-up. The secondary outcome was in-hospital death. Other events of clinical interest included arterial embolism during hospitalization, IE recurrence (>6 months after healing) and rehospitalization for acute heart failure (HF) at follow-up.

All records concerning in-hospital course, including those about surgical treatment, if any, were collected. Data were retrospectively collected from the records of ambulatory visits performed in the department by the cardiologist and the infectious diseases specialist. Whenever patient’s data were missing, we gathered information by contacting the patient himself or his/her family. When none of these options enable gathering of missing data, the hospital registers were also checked. These data were collected and gathered during the months of September and October 2017. All data were collected in a single computerized datasheet.

We achieved the information about the follow-up either through clinical visit or through telephone interview or by consulting the hospital register concerning death and the rehospitalization. When follow-up status was not available, the patient was considered lost to follow-up.

### 2.5 Statistical analysis

Continuous normally-distributed data are presented as mean ± standard deviation and compared by using the Student t-test. Continuous variables with asymmetrical distribution were reported as median and interquartile range and compared by using the Mann-Whitney U test. Categorical variables are presented as numbers and percentages and compared by using the Chi-square or the Fisher’s exact test, as appropriate. The cumulative incidence of all-cause death was estimated at different time frames using the Kaplan-Meier method and the Log-rank test was used for comparison between groups.

All the variables statistically different among groups, were tested at the univariable analysis. Binary logistic regression was used for the in-hospital outcome, whereas proportional hazard Cox regression was employed for the analysis at long-term. Furthermore, multivariable stepwise forward regressions were performed to identify a set of independent predictors of outcome. Results were reported as hazard ratios (HR) with 95% confidence intervals (95%CI). The Hosmer–Lemeshow statistic was evaluated to assess the goodness-of-fit of the logistic regression model.

Propensity score analysis was used to take into account of imbalances in the baseline characteristics of patients by exposure groups (diabetic vs. non-diabetic patients). The following covariates were included in the model according to the baseline differences between groups or to the pathophysiological association with diabetes: age, sex, hypertension, history of HF, coronary artery disease (CAD), chronic kidney disease (CKD), chronic cerebrovascular disease (CVD), prior stroke/Transient ischemic attack (TIA), peripheral artery disease (PAD), prosthetic valve and IVDUs. The propensity score model showed an adequate discrimination with area under the curve values of 0.75 and it was entered as a covariate for adjustment in all univariable and multivariable Cox regression analyses. The consistency of the results for the overall mortality at long term was also investigated in the following subgroups of clinical interest: non-IVDUs, patients aged ≤60 and >60 years. For all test, a p values <0.05 was considered statistically significant. Statistical analysis was performed by using SPSS software version 24.0 (SPSS Inc., Chicago, Illinois).

## Results

### 3.1 Study population

**Fig 1:**
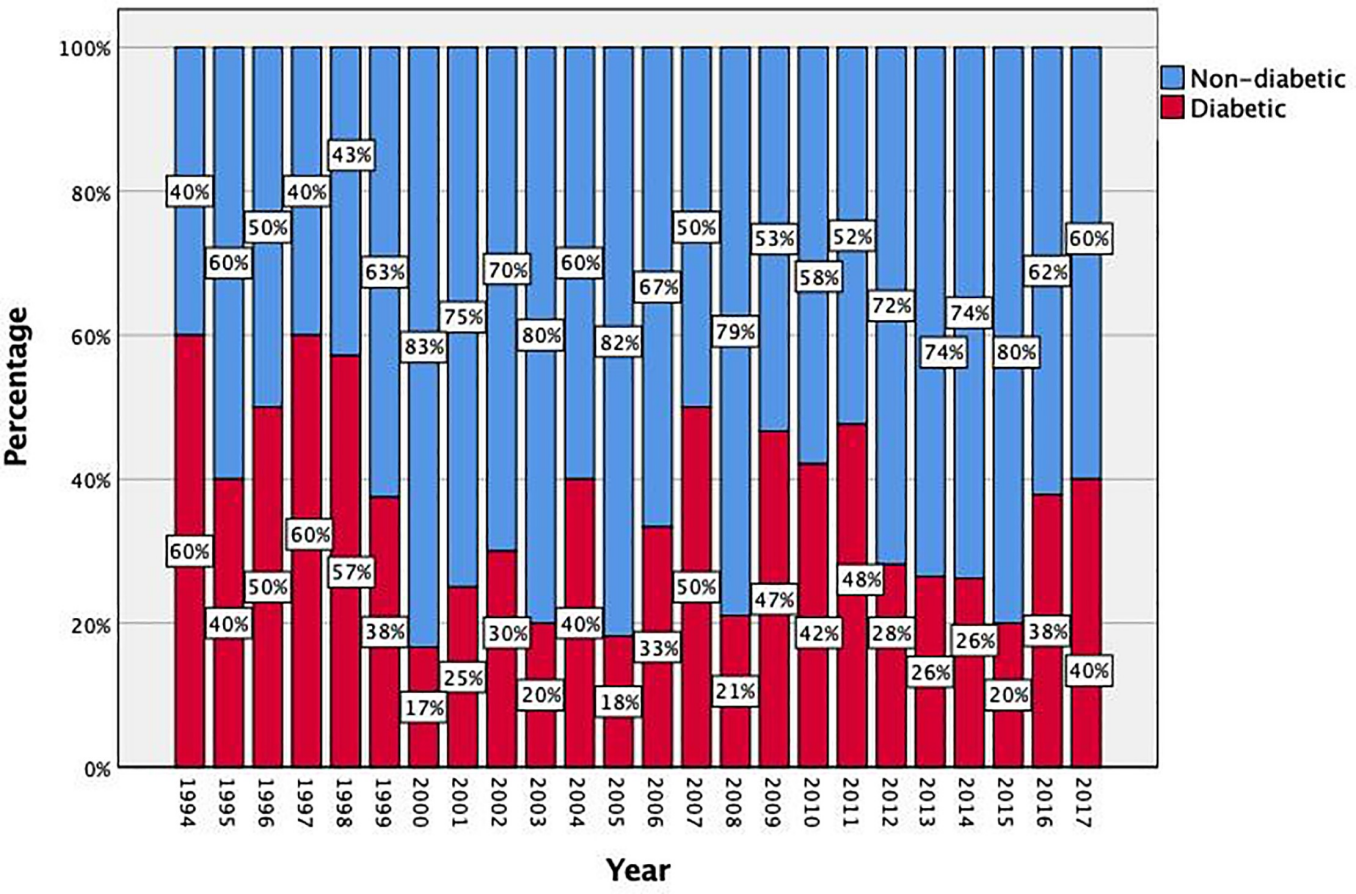
Bar graph showing the relative percentage of diabetic/non-diabetic patients over the years. The percentage of diabetic patients does not show a substantial increasing/decreasing trend during the study period.

During the observation period (1994–2017), a total of 375 patients had a definitive diagnosis of IE at our institution; DM was reported in 129 patients (34.4%). There was no secular trend in the prevalence of DM in IE over the years (Fig 1). The baseline characteristics of the overall population as well as of diabetic and non-diabetic groups, are shown in Table 1. Diabetic patients were significantly older and, as expected, showed a higher prevalence of comorbidities such as hypertension (75 vs. 54%, p<0.001), CAD (30 vs. 12%, p<0.001), CKD (50 vs. 34%, p = 0.005) and CVD (20 vs. 5%, p<0.001). Conversely, no significant difference was observed among groups with regard to IE predisposing conditions, including presence of a prosthetic valve, intracardiac devices, bicuspid aortic valve and mitral prolapse. A total of 27 IVDUs were present in the overall population, all belonging to the non-diabetic group (0 vs. 11%; p = 0.001).

**Table 1:** Clinical characteristics of the study population.

	Overall (n = 375)	Diabetic patients (n = 129)	Non-diabetic patients (n = 246)	p
Age,yrs±DS	60±16	66±12	57±17	<0.001
Male sex, n(%)	248(66)	81(62)	167(67)	0.303
Hypertension, n(%)	232(61)	97(75)	135(54)	<0.001
CAD, n(%)	70(19)	39(30)	31(12)	<0.001
History of HF, n(%)	65(17)	31(24)	34(13)	0.021
CKD, n(%)	151(40)	65(50)	86(34)	0.005
-stadium > 2, n(%)	122(32)	53(41)	69(28)	0.015
-dialysis, n(%)	34(9)	15(11)	19(7)	0.256
Liver insufficiency, n(%)	39(10)	11(8)	28(11)	0.477
-severe/cirrhosis, n(%)	16(4)	4(3)	12(7)	0.592
Dementia, n(%)	12(3)	5(4)	7(3)	0.759
COPD, n(%)	56(15)	24(18)	32(13)	0.172
CVD, n(%)	37(10)	25(20)	12(5)	<0.001
Prior stroke/TIA, n(%)	23(6)	11(8)	12(5)	0.174
PAD, n(%)	25(7)	13(10)	12(5)	0.058
Neoplasia, n(%)	30(8)	9(6.9)	21(8)	0.691
Prosthetic valve, n(%)	95(25)	60(46)	35(14)	0.618
BAV, n(%)	25(7)	6(5)	19(7)	0.285
Leaflet prolapse, n(%)	71(19)	25(19)	46(18)	0.890
PM/ICD, n(%)	39(10)	17(13)	22(9)	0.216
CVC, n(%)	44(12)	18(14)	26(10)	0.399
HIV, n(%)	6(2)	0(0)	6(2)	0.097
IVDUs, n(%)	27(7)	0(0)	27(11)	<0.001
Alcohol abuse, n(%)	2(0,5)	0(0)	2(1)	0.547
Prior IE, n(%)	25(7)	10(8)	15(6)	0.664

BAV: Bicuspid Aortic Valve; CAD: Coronary Artery Disease; COPD: Chronic Obstructive Pulmonary Disease; CKD: Chronic Kidney Disease; CVC: Catheter Venous Central; CVD: Chronic Cerebrovascular Disease; IVDUs: Intravenous Drug Users; HF: Heart Failure; HIV: Human Immunodeficiency Virus; PAD: Peripheral Artery Disease; PM/ICD: Pacemaker and/or Internal Cardiac Device; Prior hospitalization for Infective Endocarditis; TIA: Transient ischemic attack

### 3.2 Microbiology

A pathogenic microorganism was isolated in 292 of 375 patients (77.8%; 79% in the diabetic group vs. 77% in the non-diabetic group, p = 0.140). No significant differences were found with regard to the causative microorganism, although a slight increase in prevalence of the Coagulase-negative Staphylococci (CoNS) and Enterococci was observed in diabetic patients (22.5 vs. 17.9%, p = 0.337 and 16.3 vs. 11%, p = 0.193, respectively; Table 2). Oral Streptococci were prevalent in the non-diabetic group (19.5 vs 12.4%, p = 0.084).

**Table 2:** Microbiological findings.

	Overall (n = 375)	Diabetic patients (n = 129)	Non-diabetic patients (n = 246)	*p*
Staphylococcus aureus,n (%)	58(15)	17(13)	41(17)	0.453
Oral Streptococci, n(%)	64(17)	16(12)	48(10)	0.084
CoNS, n(%)	73(19)	29(22)	44(18)	0.337
Group D Streptococci, n(%)	21(6)	9(10)	12(5)	0.480
Enterococci, n(%)	48(13)	21(16)	27(11)	0.193
Candida, n(%)	8(2)	4(3)	4(2)	0.456
Others, n(%)	39(10)	14(11)	25(10)	0.860
Negative blood culture, n(%)	83(22)	27(21)	56(23)	0.140

CoNS: Coagulase Negative Staphylococci

### 3.3 Echocardiography

Overall, aortic valve was the most commonly involved, with a significantly higher prevalence in diabetic patients (57 vs. 46%, p = 0.039; Table 3). A higher, although not statistical, involvement of the tricuspid valve was observed in the non-diabetic group (15 vs. 8%, p = 0.055). Vegetations were detected more frequently in diabetic than in non-diabetic patients (91 vs. 81%, p = 0.048), without significant differences in term of size, mobility and extent (Table 3). Diabetic patients showed a higher rate of paravalvular complications (82 vs. 64%, p<0.001), including abscess, new periprosthetic leak, pseudoaneurysm and prosthetic valve dehiscence. LVEF at hospital admission was significantly lower in the diabetic than in non-diabetic patients (52±11 vs. 55±10%, p = 0.001).

**Table 3:** Echocardiographic findings.

	Overall (n = 375)	Diabetic patients (n = 129)	Non-diabetic patients (n = 246)	*p*
Valve involved				
-aortic, n(%)	188(50)	74(57)	114(46)	0.039
-mitral, n(%)	144(38)	48(37)	96(39)	0.754
-tricuspid, n(%)	46(12)	10(8)	36(15)	0.055
-pulmonary, n(%)	2(0.5)	0(0)	2(0.8)	0.305
Catheter-involving endocarditis, n(%)	43(11)	19(15)	24(10)	0.147
Valvular regurgitation*, n(%)	246(65)	81(63)	165(67)	0.406
Peri-valvular complications **, n(%)	265(77)	106(82)	159(64)	<0.001
Vegetation, n(%)	320(85)	118(91)	202(81)	0.048
Size of vegetation				
-grade 1, n(%)	96(25)	34(26)	62(25)	0.791
-grade 2, n(%)	95(25)	38(29)	57(23)	0.176
-grade 3, n(%)	56(14)	20(15)	36(15)	0.810
-grade 4, n(%)	66(17)	22(17)	44(18)	0.854
Mobility of vegetation				
-grade 1, n(%)	23(6)	8(7)	15(6)	0.382
-grade 2, n(%)	121(32)	44(31)	77(31)	0.563
-grade 3, n(%)	103(27)	41(32)	62(25)	0.168
-grade 4, n(%)	60(16)	19(15)	41(17)	0.638
Extent of vegetation				
-grade 1, n(%)	170(45)	65(51)	105(43)	0.154
-grade 2, n(%)	25(7)	4(3)	21(9)	0.045
-grade 3, n(%)	64(17)	24(19)	40(16)	0.567
-grade 4, n(%)	19(5)	6(5)	13(5)	0.790
LVEF, %±DS	54±11	52±11	55±10	0.001

* Including moderate or severe regurgitation

** Paravalvular complications includes: paravalvular abscess, valvular prolapse, periprosthetic leak, pseudoaneurysm, rupture of valvular cord, prosthetic dehiscence.

The rate of surgery was comparable among groups, whereas a trend toward higher need of catheters extraction was observed in the diabetic patients (12.4 vs. 6.9%, p = 0.074).

### 3.4 Clinical outcome

Fourteen patients were lost at follow-up (10 in the non-diabetic and 4 in the diabetic group). The remaining 361 showed a median follow-up of 24 months (7–84). The rates of all-cause death in-hospital and at long-term are reported in Table 4. Diabetic patients had a considerably poorer in-hospital survival than non-diabetic group (74 vs. 84%, p = 0.030). No difference in term of embolic events was observed among groups (33 vs. 37%, p = 0.481). There was a slight difference for spondylodiscitis, which was prevalent in non-diabetic patients (1 vs 5%; p = 0.039).

**Table 4:** Clinical events in-hospital and at follow-up.

	Overall	Diabetic patients	Non-diabetic patients	*p*
***In hospital***				
All-cause death, n(%)	73(19)	33(26)	40(16)	0.030
Embolism, n(%)	115(31)	43(33)	72(37)	0.481
-Cerebral, n(%)	45(12)	19(5)	26(7)	0.316
-Pulmonary, n(%)	31(84)	12(3)	19(5)	0.694
-Splenic, n(%)	25(67)	12(3)	13(3)	0.191
-Arterial, n(%)	37(10)	13(3)	24(6)	1.000
-Other, n(%)	7(19)	4(1)	3(1)	0.242
Spondylodiscitis, n(%)	13(3)	1(1)	12(5)	0.039
Catheter extraction, n(%)	33(9)	16(12)	17(7)	0.074
Surgery, n(%)	123(33)	39(30)	84(34)	0.443
***Long-term follow-up***				
All-cause death, n(%)	132(36)	64(51)	68(29)	0.001
Cardiac death, n(%)	78(22)	36(29)	42(18)	0.013
IE Recurrence, n(%)	49(13)	17(14)	32(13)	0.951
HF rehospitalization, n(%)	119(33)	55(44)	64(26)	0.001

IE: Infective Endocarditis; HF rehospitalization: rehospitalization for acute heart failure (HF)

**Fig 2:**
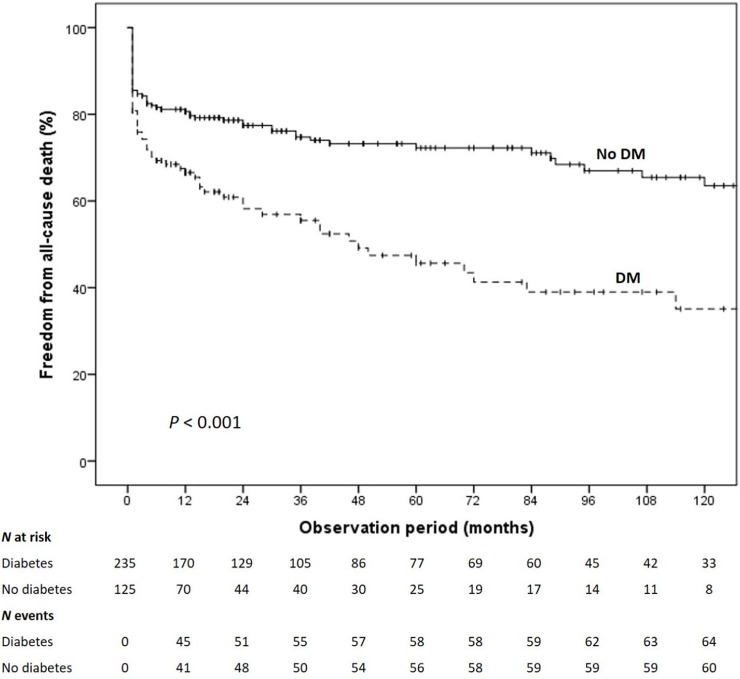
Kaplan Meier survival free from all-cause death in diabetic vs. non-diabetic patients. DM: diabetes mellitus.

During follow-up, diabetic patients showed a significantly lower survival free from all-cause death (Log-rank<0.001; Fig 2). Moreover, they also experienced a higher incidence of cardiac death (28 vs 17%, p = 0.013) and rehospitalization for acute HF (42.6 vs 25.9%, p = 0.001). There was no difference in term of IE recurrence (13 vs 13%, p = 0.951).

At logistic multivariable regression analysis, history of HF (OR = 3.12, 95%CI:1.64–5.95, p = 0.001) and low value of LVEF (OR = 0.96, 95%CI:0.93–0.98, p = 0.001) were found to be independent predictors of in-hospital mortality (Table 5).

**Table 5:** Logistic regression analysis for in-hospital mortality.

	Univariable analysis			Multivariable analysis		
	OR	CI	p	OR	CI	p
Age	1.01	0.99–1.03	0.078	-	-	-
Diabetes	1.88	1.15–3.08	0.012	-	-	-
Hypertension	1.39	0.81–2.41	0.234	-	-	-
CKD	1.64	1.00–2.69	0.050	-	-	-
CAD	1.16	0.63–2.13	0.636	-	-	-
History of HF	3.43	2.06–5.71	<0.001	3.12	1.64–5.95	0.001
CVD	1.83	0.83–4.03	0.131	-	-	-
IVDUs	1.15	0.46–2.87	0.764	-	-	-
LVEF at admission	0.95	0.93–0.97	<0.001	0.96	0.93–0.98	0.001
Aortic valve involvement	1.00	0.99–1.31	0.108	-	-	-
Vegetations	1.97	0.79–4.93	0.144	-	-	-
Surgery	0.63	0.36–1.11	0.110	-	-	-

CAD: Coronary Artery Disease; CKD: Chronic Kidney Disease; CVD: Chronic Cerebrovascular Disease; HF: Heart Failure; IVDUs: Intra-Venous Drug Users; LVEF: Left Ventricle Ejection Fraction; **OR: Odds Ratio.**

Cox multivariable analysis identified age (HR = 1.03, 95%CI:1.02–1.05, p<0.001), DM (HR = 1.75, 95%CI: 1.18–2.60, p = 0.005), history of HF (HR = 1.77, 95%CI: 1.12–2.80, p = 0.015), IVDUs (HR = 5.42, 95%CI: 2.55–11.52, p<0,001) and low LVEF (HR = 0.98, 95%CI: 0.96–0.99, p = 0.013) as independent predictors of mortality at long term (Table 6). The significant result for DM was confirmed among patients aged ≤60 years (HR = 3,50, 95%CI: 1.63–7.51, p = 0.001) and in the non-IVDUs subgroup (HR = 1.77, 95%CI: 1.20–2.63, p = 0.004; (Table 2 and Table 4). Conversely, in elderly patients (>60 years) DM was not confirmed a predictor of mortality (Table 6).

**Table 6:** Cox regression analysis for overall mortality at long-term follow-up.

	Adjusted univariable analysis			Adjusted multivariable analysis		
	Hazard Ratio	CI	*p*	Hazard Ratio	CI	*p*
Age	1.02	1.01–1.04	0.005	1.03	1.02–1.05	<0.001
Diabetes	1.83	1.23–2.71	0.003	1.75	1.18–2.60	0.005
Hypertension	0.76	0.50–1.13	0.178	-	-	-
CKD	1.70	1.15–2.51	0.007	-	-	-
CAD	0.81	0.47–1.37	0.427	-	-	-
History of HF	2.17	1.41–3.34	0.000	1.77	1.12–2.80	0.015
CVD	0.50	0.25–1.00	0.050	-	-	-
IVDUs	3.17	1.57–6.40	0.001	5.42	2.55–11.52	<0.001
LVEF at admission	0.97	0.95–0.99	0.001	0.98	0.96–0.99	0.013
Aortic valve involvement	0.87	0.61–1.24	0.438	-	-	-
Vegetations	1.15	0.65–2.01	0.625	-	-	-
Surgery	0.01	1.36–8.10	0.011	-	-	-

CAD: Coronary Artery Disease; CKD: Chronic Kidney Disease; CVD: Chronic Cerebrovascular Disease IVDUs: Intra-Venous Drug Users; HF: Heart Failure; LVEF: Left Ventricle Ejection Fraction

## Discussion

The main findings of the present study can be summarized as follows: 1) the presence of DM in patients presenting IE was associated with older age and higher presence of comorbidities like hypertension, chronic kidney disease, history of HF and CAD; 2) neither IE-predisposing conditions (except for IVDUs) nor causative agent was significantly different among groups; 3) vegetations and paravalvular complications were prevalent in diabetic than in non-diabetic patients; 4) patients with DM showed a significantly poorer outcome both in hospital and at long-term; 5) high values of LVEF are associated to a better outcome in-hospital and at long-term follow-up.

Since the prevalence of DM is growing among patients suffering with IE, the clinical impact of DM on IE is clearly a subject of interest. In fact, the epidemiology of patients affected by IE is changing through older and comorbid patients [19–20], and DM has been shown to increase the risk of IE [21]. In line with these observations, we found no difference among diabetic and non-diabetic patients in terms of IE-predisposing conditions, but diabetics were significantly older and had higher prevalence of comorbidities.

Compared to the previous report of Lin CJ et al, the clinical presentation of IE among diabetic and non-diabetic patients was almost the same in our study. This difference may be related to the dissimilar microorganism isolated and to the markedly lower age of diabetic patients observed by Lin CJ et al. (mean age: 55.9 ± 11.7 years)[22]. Moreover, after adjustment for multiple confounders with the propensity score technique, in our study DM emerged as an independent predictor of mortality at long-term follow-up, particularly in subgroup with age ≤60 years.

Few studies have investigated the prognostic effect of DM in patients with IE, reporting conflicting results. Wallace et al. and Moreno et al. reported no differences in term of in-hospital mortality among diabetic and non-diabetic patients [23–24]; however, these studies included a limited number of patients with DM (14 and 13, respectively), which may have affected the generalizability of their results. Conversely, three studies involving a greater proportion of diabetic patients are consistent with our findings, showing that diabetic patients experienced a significantly higher in-hospital mortality [25–27]. Moreover, Duval et al. reported a significantly higher mortality during hospitalization in diabetic patients requiring insulin-treatment, but not in those on oral antidiabetic therapy [28].

In our report, we found that IE is more aggressive in patients with DM, with a more severe valve involvement and a higher rate of anatomic complications. These features led to higher rates of HF, embolic events and increased mortality, which severely worsened the in-hospital outcome of these patients, especially in the population aged ≤60 years. Although DM was associated to a higher in-hospital mortality in the univariable analysis, it could not predict outcome in the multivariable model including history of HF, suggesting that the more severe clinical presentation occurring in diabetics, and not the diabetic status *per se*, plays a pivotal role leading to poor early prognosis in these patients. On the contrary, we did not observe significantly differences in therapeutic strategies (surgery/conservative) between the two groups, and surgical treatment was not a predictor of outcome in the regression analysis, suggesting that differences in therapeutic strategies between diabetic and non-diabetic patients did not affect our results.

Previous reports have demonstrated that, compared with age- and sex-matched general population, patients surviving a first episode of IE have a significantly reduced survival, mainly because of IE recurrences and development of HF [29]. In this respect, in our study there was no difference about IE recurrence in the two groups, whereas the rate of rehospitalization for worsening HF was statistically higher in diabetic patients. Therefore, investigation of predictors of adverse outcome appears to be pivotal to identify high-risk patients that may beneficiate from a stricter clinical control. Importantly, we found that DM was able to predict mortality in patients with IE at long-term follow-up, independently from comorbidities like CKD or CAD as well as from clinical presentation and echocardiographic features of the index IE-episode, which were balanced in the two groups by using the propensity score model. The prognostic value of DM was not confirmed in >60 years subgroup patients, in which the age and the history of HF resulted independent predictors of mortality, confirming the results of previous studies [20–30].

Contrary to what observed in several scientific reports, we did not observe a more frequent S. aureus- aetiology in diabetic compared with non-diabetic patients [25–26]. Nevertheless, it has to be noted that the prevalence of S. Aureus is also related to clinical characteristics other than DM, like female sex, CKD in dialysis and health care associated infection [28–29;31]; thus, the different results may derive from the distribution of such characteristics between diabetic and non-diabetic patients. Moreover, there was a slight albeit not significant difference between the diabetic and not diabetics patients about the isolation of the CoNS and Enterococci, emerging microorganisms in the scenario of IE [24;32]. Interestingly, the CONS have been historically mostly associated to infection of prosthetic valves, whereas nowadays they are currently found also in native valves and in device-related IE, especially in patients with several comorbidities [33–34].

### Study limitations

Our study has several limitations. First, although our population is one of the largest cohorts of IE-patients in the current scientific literature, our data have been collected by a single tertiary center and thus the epidemiology of the enrolled patients is limited to a restricted geographical area. Second, our study is limited by its retrospective and observational nature, that may translate in potential statistical bias. Third, despite we used the propensity score technique to adjust for baseline patient-related variables, we cannot exclude a residual selection bias secondary to other concealed confounders. Fourth, data regarding anti-diabetic therapy as well as about glycemic control are lacking, thus we cannot extrapolate any information regarding the potential effect of glycemic values or insulin-dependent DM on IE-features and patients’ outcome.

## Conclusions

In patients with IE, DM is associated to a higher prevalence of vegetations, anatomic complications and a more impaired LVEF, and show a significantly higher mortality both in hospital and at long-term. In this study, DM was also an independent predictor of mortality after discharge, suggesting caution in the clinical management of this high-risk category during follow-up.
